# Relationship between Forward Head Posture and Tissue Mechanosensitivity: A Cross-Sectional Study

**DOI:** 10.3390/jcm9030634

**Published:** 2020-02-27

**Authors:** Patricia Martinez-Merinero, Susana Nuñez-Nagy, Alexander Achalandabaso-Ochoa, Ruben Fernandez-Matias, Daniel Pecos-Martin, Tomas Gallego-Izquierdo

**Affiliations:** 1 Department of Physiotherapy, Faculty of Biomedical and Health Sciencies, Universidad Europea de Madrid, 28670 Madrid, Spain; 2 Research Institute of Physiotherapy and Pain, University of Alcalá, 28805 Madrid, Spain; 3 Department of Physical Therapy, University of Alcalá, 28871 Madrid, Spain; 4 Department of Health Sciences, University of Jaén, 23071 Jaén, Spain; 5 Physiotherapy and Pain Group, University of Alcalá, 28871 Madrid, Spain

**Keywords:** forward head posture, mechanosensitivity, pressure pain threshold, neck pain

## Abstract

The relationship between forward head posture (FHP) and neck pain is not clear. FHP could possibly increase the mechanosensitivity of cervical tissues, which could lead to the development of pain depending on the adaptation capability of the central nervous system. The purpose of this study was to analyse the influence of FHP in the mechanosensitivity of articular, muscular, and neural tissues related to the cervical spine. The pressure pain threshold was bilaterally measured in different muscles and nerves and the second cervical vertebrae. The cervical spine’s range of movement was also examined. The measurements were obtained from people with (n = 32) and without (n = 64) FHP. The analyses included a 2-by-2 mixed analysis of variance (ANOVA), pairwise comparisons with Bonferroni correction, and point-biserial correlation coefficients. Subjects with FHP showed a less pressure pain threshold (PPT) in all locations except for the upper trapezius and scalenus medius muscles. They also showed less extension and right-rotation range of motion. There was no association between FHP, neck pain, disability, and headache. Nevertheless, more research is needed to evaluate the relationship between FHP, tissue mechanosensitivity, and neck pain.

## 1. Introduction

The musculoskeletal system is a biological system composed of structures that are closely related to each other and work as a mechanical unit [1]. Among these structures, the cervical spine is frequently evaluated for postural alterations. For example, forward head posture (FHP) [2] refers to an advanced position of the head relative to the shoulders [3]. It has been suggested that FHP may impose a great mechanical demand on cervical tissues and could lead to the development of pain [4]. However, there is much controversy in the literature, with some authors having found an association between FHP and neck pain [5,6], while others have not observed an association [2,7,8].

Free nerve endings are capable of generating inflammation when exposed to a high-intensity mechanical stimulus or low-intensity stimulus that is repeated or maintained over time [9]. FHP could lead to an increase in the stress imposed on articular, muscular, and neural tissues of the neck or upper limb, which could lead to the development of pain depending on the tolerance and adaptation capability of the central nervous system [10]. It has been shown that some postural alterations like a depressed scapula can alter the mechanosensitivity of different tissues, thus decreasing their mechanical stress tolerance without evoking a nociceptive response [11]. Recently, it was found that FHP is not associated with the pressure pain threshold (PPT) measured in the upper trapezius (UT) and the articular pillars of C5–C6 in asymptomatic college students, but symptomatic subjects with FHP tended to have high PPT in the right UT [12]. Furthermore, Moustafa et al. [13] showed that a specific intervention meant to reduce FHP has a significant positive effect on neck pain, neck disability, and cervical angle at one-year follow up.

It is possible that FHP increases the mechanosensitivity of some cervical tissues and thus acts as a moderator of the relationship between FHP and neck pain. The objective of this study was to analyze the influence of FHP on the mechanosensitivity of articular, muscular, and neural tissues related to the cervical spine, as well as its influence on the cervical range of movement.

## 2. Methods

### 2.1. Study Design

This cross-sectional study was conducted according to the recommendations of the *Strengthening the Reporting of Observational Studies in Epidemiology* (*STROBE*) [14]. Ethical approval was obtained from the Ethical Committee of University Cardenal Herrera Oria, San Pablo Ceu, Valencia, Spain (CEI 15/002). The study was also conducted according to the Declaration of Helsinki.

### 2.2. Subjects

A convenience sample of college students was recruited through announcements at the University of Alcala (Spain). Before participation, all subjects signed a consent form. For the descriptive analysis of the sample, information on age, height, weight, sex, and body mass index (BMI) was collected. Subjects were included in the study if they were older than 18 years. They were excluded if they presented severe cervical arthrosis, disc herniation, neurologic symptoms, straightening of the cervical lordosis, trauma in the past year, or any systemic diseases.

### 2.3. Sample Size

The sample size was calculated based on the main between-subjects’ effect (FHP) of a 2-by-2 mixed analysis of variance (ANOVA). The effect size was estimated to be 0.25, with a correlation between repeated measures of 0.50, 80% power, and α value of 0.05. According to the sample size calculation, 96 subjects (32 cases and 64 controls) had to be recruited.

### 2.4. Measurements

#### 2.4.1. Pain and Disability

Neck pain intensity during the last week was measured with a visual analogue scale (VAS), a reliable tool (*r* = 0.94), where 0 represents no pain, and 10 represents the worst pain imaginable [15]. The presence of chronic neck pain and headache lasting more than 3 months was also registered separately as dichotomic variables (yes, no). Disability was measured with the neck disability index (NDI), a valid and reliable (ICC = 0.98) questionnaire that was transculturally adapted from English to Spanish (Spain) in 2010. An index of 0 represents no disability, and 50 represents total disability [16]. The raters that measured the dependent variables were blinded to the presence of pain, pain intensity, and disability.

#### 2.4.2. Cranio-Cervical Angle

Postural Assessment Software (PAS) was used for the measurement of the cranio-cervical angle (CCA) [17,18]. Photographs were taken with a reflex camera (Nikon Model D5300 SLR, Tokyo, Japan) that was fixed with a tripod and placed three meters away from the subject [19]. The subject was standing and was told to maintain a relaxed natural position. Only one photograph per subject was taken.

Two markers were placed on the tragus of the ear and the spinous apophysis of the seventh cervical vertebrae (C7). Two lines were traced for the measurement of the CCA. The first one was a horizontal line that crossed the C7 marker, and the second one was an oblique line that linked up the C7 and tragus markers. The CCA was measured as the angle between the two lines [20,21,22,23]. This procedure has shown good reliability with an intra-class correlation coefficient (ICC) of 0.98 and good construct validity in comparison to radiography with a Pearson correlation coefficient of 0.89 [24].

A cut-off point of 50° was used for the classification of the subjects into control or FHP groups. Subjects with a CCA of 50° or more were classified as controls, and subjects with a CCA below 50° were classified as cases with FHP [23]. The photographs and CCA measurements were obtained by evaluator 1, who is a physiotherapist with more than 10 years of experience in musculoskeletal pain assessment and treatment.

#### 2.4.3. Cervical Range of Movement

After the measurement of the CCA, the assessment of the cervical range of movement was also performed by evaluator 1 using a cervical goniometer device (CROM, Performance Attainment Associates, St. Paul, MN). The CROM is made up of three goniometers and a magnet system and has been shown to be a valid and reliable tool (ICCs > 0.90) [25]. The subject was seated in a chair in an upright position during the CROM measurements and was told to move as far as he/she could. Three measurements were taken for each motion (flexion, extension, right and left rotation, and right and left side bending) with a 30-s rest period in between them. The mean of the three measurements was used for the statistical analysis.

#### 2.4.4. Pressure Pain Threshold

The tissues’ degree of mechanosensitivity was evaluated with PPT, which was measured with a hand-held algometer (Wagner Force Dial, Model FDK 20), which has a 1-cm^2^ head that records pressure in kg/cm^2^ [26]. The pressure was increased by 1 kg per second, and the subject was told to indicate when the sensation changed from pressure to pain. Three measurements were taken in each location with a 30-s rest period in between them. The mean of the three measurements was used for the statistical analysis.

Evaluator 2, a physiotherapist expert in manual therapy with more than 10 years of experience, measured PPT bilaterally in the following muscles: The upper trapezius (UT), levator scapulae (LS), splenius capitis (SC), sternocleidomastoid (SCM), and scalenus medius (SM). The muscle belly was palpated to locate the most mechanosensitive point where PPT were measured. The measurement of PPT with a hand-held algometer in the local muscles of subjects with neck pain has been shown to have good reliability, with an ICC of 0.87 to 0.89 [27].

Evaluator 3, a physiotherapist expert in manual therapy with more than 10 years of experience, conducted the PPT measurements bilaterally on the median, radial, and ulnar nerves and on the posterior aspect of the articular facets of the second cervical vertebrae (C2). The nerves were evaluated in the locations described by Sterling et al. [28], which have shown good reliability, with an ICC of 0.92 to 0.97. The median nerve was identified in the cubital fossa medial to and adjacent to the tendon of the biceps. The ulnar nerve was identified in the ulnar groove of the elbow with the shoulder with 90° of abduction and external rotation and the elbow with 90° of flexion. The radial nerve was identified in the lateral intermuscular septum between the medial and lateral heads of the triceps [28]. All three evaluators were blinded to each other’s measurements.

### 2.5. Statistical Analysis

A normal distribution of the data was assumed based on the central limit theorem as both groups had more than 30 subjects. For the differences between groups of the bilaterally measured quantitative variables, a 2-by-2 mixed model ANOVA was conducted with FHP (yes, no) as a between-subjects’ factor and side (right, left) as a within-subjects’ factor. Post hoc pairwise comparisons and the differences between groups in the non-bilaterally measured variables were analyzed using student *t*-tests with Bonferroni correction. Differences between groups in the categorical variables were analyzed with the chi-squared test.

For the correlation analysis between FHP (yes, no) and quantitative variables, a point-biserial correlation coefficient (*r*_pb_) was calculated. For categorical variables, Cramer’s *V* was used. Cohen’s *d* was used as an estimator of the effect size for the between-group differences in the quantitative variables:

*d* = 2*t*/√*g*. (1)

All the analyses were conducted using the statistical software SPSS v22.00 (SPSS Inc., Chicago, IL, USA). An α level of 0.05 and 95% confidence intervals (CIs) were assumed for all analyses.

## 3. Results

**Figure 1:**
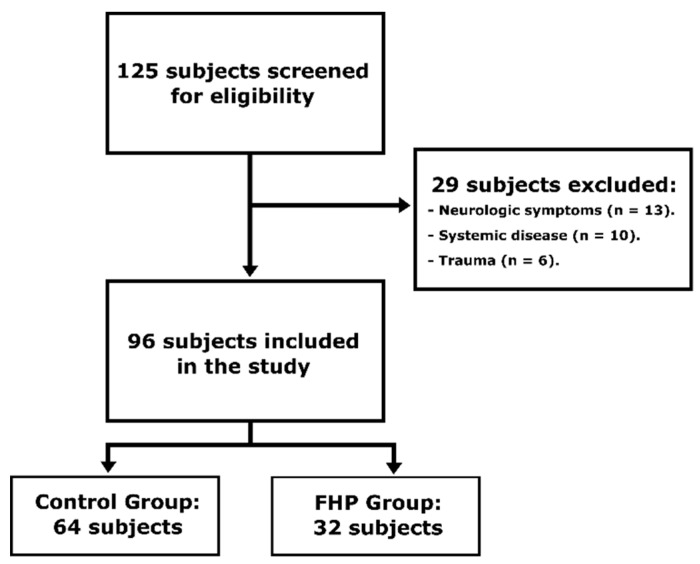
Flow diagram of subjects.

The final sample was composed of 96 subjects (Figure 1): 64 controls with a mean age of 19.48 (SD, 1.96) years and 32 cases with a mean age of 20.53 (SD, 2.96) years. The mean CCA was 52.30° (3.00°) in the control group and 44.63° (4.20°) in the FHP group. Demographic characteristics of the subjects are presented in Table 1.

### 3.1. Pain and Disability

There were no statistically significant differences between groups in the frequency of cervical pain or headaches (Table 2). There were also no statistically significant differences between groups in pain intensity during the last week or disability (Table 2).

### 3.2. Cervical Range of Movement

There was a statistically significant difference between groups in cervical extension range of motion (*d* = 0.56, *p* < 0.05). For cervical rotation range of motion, the 2-by-2 mixed model ANOVA revealed a significant main effect for FHP (F = 4.77, *p* = 0.03) and side (F = 23.25, *p* < 0.001), as well as a significant FHP-by-side interaction (F = 27.73, *p* < 0.001). For cervical side bending range of motion, the 2-by-2 mixed model ANOVA revealed a non-significant main effect for FHP and side, as well as non-significant FHP-by-side interaction. Post hoc analysis revealed significant differences between groups in right rotation (*d* = 0.85, *p* < 0.001). The results of all other pairwise comparisons were not significant (Table 3). There was a significant correlation between FHP and cervical extension and right rotation range of motion (*p* < 0.05). All other correlations were not significant (Table 3).

### 3.3. Muscle Mechanosensitivity

The 2-by-2 mixed model ANOVAs revealed a significant main effect for FHP in the PPT of the LS (F = 6.30, *p* = 0.01), SC (F = 4.54, *p* = 0.04) and SCM (F = 15.94, *p* < 0.001) muscles but not for the UT and SM muscles. There was no significant main effect for side and no significant FHP-by-side interaction in any muscle. The post hoc comparison results are presented in Table 4. There was a significant correlation between FHP and PPT of the right and left LS muscles, right and left SC muscles, and right and left SCM muscles (*p < 0.05)*. All the other correlations were not significant (Table 4).

### 3.4. Neural and Articular Mechanosensitivity

The 2-by-2 mixed model ANOVAs revealed a significant main effect for FHP in the PPT of the median (F = 12.44, *p* < 0.001), radial (F = 10.04, *p* = 0.002), and ulnar (F = 6.78, *p* = 0.01) nerves, as well as the PPT of C2 (F = 20.61, *p* < 0.001). There was also a significant main effect for side in the PPT of the median (F = 5.83, *p* = 0.02) and ulnar nerves (F = 5.20, *p* = 0.03). Finally, there was a significant FHP-by-side interaction for the PPT of the median nerve (F = 6.30, *p* = 0.01).

There was no significant main effect for side in the PPT of the radial nerve and C2. There was also no significant FHP-by-side interaction in the PPT of the radial and ulnar nerves or C2. The post hoc comparison results are presented in Table 5. There was a statistically significant correlation between FHP and the PPT of the right (*p* < 0.001) and left (*p* < 0.05) median nerves, right (*p* < 0.05) and left (*p* < 0.05) radial nerves, left ulnar nerve (*p* < 0.05), and right (*p* < 0.001) and left (*p* < 0.05) C2 articular facets. There was no significant correlation between FHP and PPT of the right ulnar nerve (Table 5).

## 4. Discussion

### 4.1. Relationship between FHP, Pain, and Disability

The average CCA was 52.3° in the control group and 44.6° in the FHP group, which are rather similar to the ones observed by Shaghayegh et al. [29] in 2016. According to the classification proposed by Salahzadeh et al. [30], the FHP group in the present study had severe-moderate FHP. There was no association between FHP and neck pain. The literature on this topic is controversial. Some studies have not found an association between FHP and pain [2,8], while others have found such an association [5,6]. However, while the subjects in the present study had mechanical cervical pain of nonspecific origin, the ones in the study of Yip et al. [5] had neck pain and numbness/paresthesia sensations in the upper limb, and the ones examined by Diab et al. [6] had cervical spondylosis and radicular pain. These discrepancies between studies could be due to the fact that chronic pain subjects show more complex interactions, with emotional distress hindering their pain modulation capability. We do not know if this result applies to subjects with acute neck pain, in which mechanical aspects are more important [31]. Headache was not found to be associated with FHP. There are controversies in the literature on this topic as well, with some studies finding an association [32,33] while others have not [34,35]. According to Oliveira et al. [36], it is possible that these discrepancies between the results of the different studies may be due to methodological issues, like the absence of blinding, differences in CCA measurement procedures, absence of sample size calculation, or heterogeneity in the studied popula1tions. All these issues make it difficult to compare the results between different studies.

There was no association between FHP and disability measured with NDI. These results are contrary to the ones obtained by Yip et al. [5], but these researchers used a different questionnaire, the Northwick Park Test. Kim et al. [37] also found an association between FHP and disability, but they did not examine whether the subjects had neck pain and did not include a control group.

### 4.2. Relationship between FHP and Cervical Range of Movement

Descriptive analyses showed less range of movement of the cervical spine in all planes in subjects with FHP in comparison to the controls. However, these differences were only statistically significant for the extension and right rotation range of motion. The differences between groups in right rotation and left rotation range of motion could be explained by the fact that the location of pain in subjects with FHP and neck pain was more prevalent on the right side, as the major differences in rotation range of motion between sides were observed in these subjects. However, this did not happen in the control group, where the pain location was mixed. Therefore, pain location could have produced the observed differences between the controls and subjects with FHP (the decrease in cervical spine range of motion in subjects with FHP).

Quek et al. [38] found that subjects with FHP between 30 and 50 years old had less flexion and total rotation range of motion. These findings have also been observed in subjects with other pain syndromes like tunnel carpal tunnel syndrome [39], tension headache [33], and migraine [32]. The negative results of this study could be due to the age of the subjects as our sample was very young, so more time could be necessary for FHP to produce a reduction in the range of motion. The fact that only two movements showed statistically significant differences in the present study implies that more research is needed to clarify the relationship between FHP and the range of movement of the cervical spine.

### 4.3. Relationship between FHP and Tissue Mechanosensitivity

We found a significant increase in mechanosensitivity in the FHP group in all locations except for both UT muscles, both SM muscles, and the right ulnar nerve. The effect sizes were small to moderate, which should be taken into account as FHP could influence tissue mechanosensitivity, although not to a great extent. These results could be because FHP could increase the compressive forces on the cervical apophyseal joints and the mechanical stress of the shoulder and neck muscles [40], which could subsequently lead to nerve sensitization [41]. There is only one published study that has investigated the influence of FHP on PPT. Pacheco et al. [12] found that subjects with FHP and subclinical neck pain had higher PPT in the right UT. Contrary to the results of the present study, Pacheco et al. [12] found no association between FHP and PPT in their healthy group.

Repetitive mechanical stress over time could promote the appearance of algogenic substances that could lead to tissue hyperalgesia [41]. Some authors have suggested that postural alterations could play a role in the development of pain through an increase in tissue mechanosensitivity [11,42]. FHP could potentially lead to a situation of tissue hyperalgesia, as shown in the present study by a reduction in PPT, which could trigger neck pain or headache depending on the adaptation capability of the central nervous system.

### 4.4. Limitations

One important limitation of this study is the specificity of the sample (college students), so it is difficult to make inferences about the results in older populations. Another important limitation is the unbalanced design of the study. Unbalanced designs can diminish the power of ANOVA to detect an effect and lead to an increase in false-negative results. However, false-positive results are not increased with unbalanced designs, so the statistically significant results of the study can still be trusted.

Although the procedures used for the assessment of the variables have shown good reliability in previous studies, the reliability of the evaluators of the present study was not assessed. Daffin et al. [43] recently showed that people who show FHP in photogrammetric analyses have different underlying cervical spine shapes in radiological analyses. This limits the conclusions of the present study as we do not know the actual differences in the cervical spine shape between groups. Finally, although it has been observed that subjects with FHP have an increase in tissue mechanosensitivity, due to the cross-sectional design of the study, it cannot be stated that a cause–effect relationship exists between FHP and an increase in tissue mechanosensitivity.

## 5. Conclusions

Based on the results of the present study, it seems that subjects with FHP have an increase in tissue mechanosensitivity and a decrease in cervical range of motion. However, it seems that FHP is not associated with the presence of neck pain, headache, or disability. Further research is needed to clarify the implications of tissue mechanosensitivity as an effect moderator in the relationship between FHP and cervical pain.

## Figures and Tables

**Table 1:** Demographic characteristics of the subjects (n = 96).

Characteristic, Mean (SD)	Control Group (n = 64)	FHP Group (n = 32)	*p*-Value
Age, years	19.48 (1.96)	20.53 (2.96)	0.657
Height, cm	172.19 (9.29)	169.44 (9.64)	0.181
Weight, kg	63.48 (9.98)	63.95 (13.65)	0.863
BMI, kg/m^2^	21.17 (2.09)	21.93 (2.90)	0.200
CCA, degrees	52.28 (3.00)	44.63 (4.20)	<0.001
Sex, women; n (%)	38 (59.38)	21 (65.63)	0.55

Abbreviations: SD, standard deviation; FHP, forward head posture; BMI, body mass index; CCA, cranio-cervical angle.

**Table 2:** Between group differences in pain and disability.

Variable	Control Group (n = 64)	FHP Group (n = 32)	Effect Size
Cervical pain, n (%)	22 (34.38)	8 (25)	0.11 ^a^
Headache, n (%)	9 (14.06)	4 (12.5)	0.01 ^a^
VAS, mean cm (95% CI)	1.85 (1.17, 2.53)	1.35 (0.40, 2.30)	
Difference VAS, mean (95% CI)	0.50 (−0.65, 1.66)		0.09 ^b^
NDI, mean (95% CI)	5.89 (3.52, 8.26)	4.44 (0.96, 7.92)	
Difference NDI, mean (95% CI)	1.45 (−2.70, 5.60)		0.07 ^b^

^a^ Cramer’s *V*, ^b^ Cohen’s *d*. Abbreviations: FHP, forward head posture; VAS, visual analogue scale; CI, confidence interval; NDI, Neck Disability Index.

**Table 3:** Between group differences in cervical range of movement.

Variable, ° Mean (95% CI)	Control Group (n = 64)	FHP Group (n = 32)	Differences	*d*	*r_pb_*
Flexion	62.55 (60.35, 64.74)	60.00 (56.57, 63.43)	2.55 (−1.33, 6.43)	0.27	0.13
Extension	78.81 (76.54, 81.08)	72.56 (67.82, 77.30)	6.25 (1.69, 10.81) ^†^	0.56	0.27 ^†^
Side bending (R)	58.89 (55.34, 62.40)	53.33 (47.66, 58.28)	5.56 (−0.51, 11.67)	0.37	0.18
Side bending (L)	58.30 (54.72, 61.88)	54.84 (49.78, 59.91)	3.45 (−2.75, 9.65)	0.23	0.11
Rotation (R)	57.27 (53.96, 60.58)	45.38 (40.69, 50.06)	11.89 (6.16, 17.62) ^‡^	0.85	0.39 ^†^
Rotation (L)	56.75 (53.44, 60.06)	57.09 (52.41, 61.78)	−0.34 (−5.39, 6.08)	0.02	0.01

^†^ Statistically significant (*p* < 0.05), ^‡^ Statistically significant (*p* < 0.001). Abbreviations: °, degree; CI, confidence interval; FHP, forward head posture; *d*, Cohen’s *d*; *r*_pb_, point-biserial correlation coefficient; R, right; L, left.

**Table 4:** Between group differences in muscles’ pressure pain thresholds.

PPT kg/cm^2^, Mean (95% CI)	Control Group (n = 64)	FHP Group (n = 32)	Differences	*d*	*r_pb_*
UT (R)	2.75 (2.57, 2.93)	2.49 (2.24, 2.75)	0.26 (−0.05, 0.57)	0.34	0.17
UT (L)	2.73 (2.54, 2.92)	2.57 (2.30, 2.83)	0.16 (−0.16, 0.49)	0.21	0.10
LS (R)	2.60 (2.43, 2.77)	2.19 (1.94, 2.43)	0.42 (0.12, 0.71) ^†^	0.61	0.29 ^†^
LS (L)	2.59 (2.40, 2.78)	2.24 (1.98, 2.51)	0.35 (0.02, 0.67) ^†^	0.43	0.22 ^†^
SC (R)	2.43 (2.27, 2.59)	2.10 (1.88, 2.32)	0.33 (0.06, 0.59) ^†^	0.47	0.22 ^†^
SC (L)	2.39 (2.20, 2.57)	2.09 (1.86, 2.31)	0.30 (0.01, 0.58) ^†^	0.37	0.20 ^†^
SM (R)	1.99 (1.86, 2.11)	1.85 (1.67, 2.02)	0.14 (−0.08, 0.36)	0.27	0.13
SM (L)	1.93 (1.82, 2.05)	1.82 (1.66, 1.98)	0.11 (−0.09, 0.31)	0.23	0.11
SCM (R)	1.90 (1.78, 2.02)	1.52 (1.36, 1.69)	0.38 (0.18, 0.58) ^‡^	0.77	0.36 ^‡^
SCM (L)	1.89 (1.77, 2.00)	1.56 (1.39, 1.72)	0.33 (0.13, 0.53) ^†^	0.67	0.32 ^†^

^†^ Statistically significant (*p* < 0.05), ^‡^ Statistically significant (*p* < 0.001). Abbreviations: PPT, pressure pain threshold; CI, confidence interval; FHP, forward head posture; R, right; L, left; *d*, Cohen’s *d*; *r*_pb_, point-biserial correlation coefficient; UT, upper trapezius; LS, levator scapulae; SC, splenius capitis; SM, scalenus medius; SCM, sternocleidomastoid.

**Table 5:** Between group differences in the pressure pain threshold of the nerves and C2.

PPT kg/cm^2^, Mean (95% CI)	Control Group (n = 64)	FHP Group (n = 32)	Differences	*d*	*r_pb_*
Median nerve (R)	3.03 (2.82, 3.24)	2.32 (2.02, 2.61)	0.72 (0.35, 1.08) ^‡^	0.80	0.37 ^‡^
Median nerve (L)	2.79 (2.60, 2.98)	2.32 (2.06, 2.59)	0.46 (0.14, 0.79) ^†^	0.58	0.28 ^†^
Radial nerve (R)	3.12 (2.88, 3.33)	2.44 (2.12, 2.76)	0.67 (0.27, 1.06) ^‡^	0.69	0.33 ^†^
Radial nerve (L)	3.01 (2.78, 3.25)	2.47 (2.14, 2.80)	0.55 (0.14, 0.95) ^†^	0.55	0.27 ^†^
Ulnar nerve (R)	3.08 (2.81, 3.34)	2.64 (2.34, 2.95)	0.41 (0.02, 0.79) ^†^	0.40	0.20
Ulnar nerve (L)	3.04 (2.77, 3.31)	2.44 (2.24, 2.65)	0.59 (0.26, 0.91) ^†^	0.63	0.30 ^†^
C2 (R)	2.95 (2.79, 3.12)	2.28 (2.10, 2.47)	0.69 (0.40, 0.98) ^‡^	0.98	0.44 ^‡^
C2 (L)	2.73 (2.58, 2.89)	2.30 (2.07, 2.52)	0.44 (0.16, 0.72) ^†^	0.64	0.31 ^†^

^†^ Statistically significant (*p* < 0.05), ^‡^ Statistically significant (*p* < 0.001). Abbreviations: PPT, pressure pain threshold; CI, confidence interval; FHP, forward head posture; *d*, Cohen’s *d*; *r*_pb_, point-biserial correlation coefficient; R, right; L, left; C2, second cervical vertebrae.
